# Could snort production reflect comfort in horses kept outdoors? A first study

**DOI:** 10.1007/s00114-026-02113-3

**Published:** 2026-06-30

**Authors:** Martine Hausberger, Lucas Matthieu, Maëlle Delbos, Margarita Briseño-Jaramillo, Laurence Wimel, Séverine Henry, Alban Lemasson, Clémence Lesimple

**Affiliations:** 1 Department of Zoology and Entomology, Rhodes University, Makhanda, South Africa; 2 Centre Intégratif de Neuroscience et Cognition, Université de Paris-Cité, UMR CNRS 8002, Paris, France; 3 Centre d’étude en éthologie et cognition, Université de Rennes, Université de Caen-Normandie, UMR CNRS 6552, Paimpont, France; 4 Instituto de Neuroetología, Universidad Veracruzana, Xalapa, Mexico; 5 IFCE, Station Expérimentale de Chamberet, Chamberet, France; 6 Ecole Supérieure des Agricultures (ESA), USC URSE – Unité de Recherche sur les Systèmes d’Elevage - Ecole Supérieure des Agricultures, Angers, France

**Keywords:** Weather conditions, Snorts, Positive emotions, Unvoiced signals, Temperature, Housing

## Abstract

The welfare of free-roaming, captive wild or domestic animals housed outdoors is often questioned. How subjects perceive climatic conditions is not always well detected as it is highly subjective, depending on species, age and experience. Hence, decisions on housing rely mostly on human anthropomorphic perception. In particular, indicators of comfort and positive emotions remain poorly documented. Acoustic signals, however, offer a promising window into animals’ internal states. In the present study, we hypothesized that acoustic signals could be used to identify positive or negative perceptions of environmental conditions in horses. Horses possess a diverse acoustic repertoire and the snort produced by nostril vibration, has been identified as a likely indicator of calm, positive emotions. Over 3 weeks in early spring, acoustic production and time budget were recorded for 20 adult horses living outdoors on pasture. Meteorological conditions and pasture composition were monitored simultaneously. Results showed that snorts were the most frequent acoustic signal and that their production varied considerably across observation sessions. Snort production was highly correlated with temperature: horses produced more snorts and showed more relaxed behaviours when the temperature ranged between 15 and 20 °C. In contrast, lower temperatures around 5–8 °C were associated with more “static” behaviours (reflecting energy conservation) and less snorts. Finally, snorts were more frequent immediately after horses entered a new pasture with higher food diversity than just before leaving, when food diversity had become more restricted. These findings suggest that acoustic production may help identify the subjective perception that animals have from their environment, allowing more individualized and adapted management practices.

**Supplementary Information:**

The online version contains supplementary material available at 10.1007/s00114-026-02113-3.

## Introduction

While most animal welfare studies converge in promoting life conditions for domestic and captive wild animals that come as close as possible to their natural environment, there is still some resistance to expose animals to outdoor housing conditions because of the associated weather conditions such as low or high temperature, rain or wind (e.g. Lesimple et al. 2020). The welfare of feral animals is questioned for the same reasons (Górecka-Bruzda et al. 2020).

On the one hand, many horse owners keep their horses indoors for fear of adverse weather conditions, often based on subjective anthropomorphic judgements (e.g. Hausberger et al. 2021). On the other hand, there is a growing tendency for horse owners to keep their horses outdoors for all or part of the time, even in harsh climatic conditions (Hartmann et al. 2015; Mejdell et al. 2020). This raises practical questions, such as whether they should stay outside whatever the weather conditions, what type of shelter they need and/or whether they should wear a rug (Mejdell et al. 2016, 2019). Horses, like other ungulates are homeothermic (warm blooded) and endothermic (producing heat by the body) (Sjastaad et al. 2016). Like red deers (*Cervus elaphus*) or ibexes (*Capra ibex*), they may reduce their metabolic rate in cold winter (Brinkman et al. 2014). Studies, based on experimental settings, have aimed at defining horses’ thermoneutral zone, i.e. « the span in ambient temperature within which the animal can keep the core body temperature stable, using physiological regulatory mechanisms only » (Mejdell et al. 2020) or « the zone of least thermoregulatory effect » (Hillman 2009), i.e. of thermal comfort. Morgan (1998) proposed a thermoneutral zone between 5 and 25 °C in horses. However, different studies have shown that thermal comfort is highly dependent upon a variety of parameters such as individual characteristics (e.g., age, breed, acclimatization) and environmental factors (e.g., food resources, humidity, wind or solar radiation) (reviewed in Mejdell et al. 2020). For example, Cymbaluk & Christenson (1989) have found a low critical temperature (LCT) of −15 °C in cold-adapted Quarter Horses. Consequently, this zone of thermal comfort is difficult to assess under non-experimental settings. Some behavioural changes can be observed when animals are facing adverse climatic conditions: in extreme cold, Icelandic horses may stand and aggregate with other horses (Ingólsdóttir & Sigurjónsdóttir 2008) and Przewalski horses may reduce their metabolism rate by reducing activity (Arnold et al. 2006). According to Langlois (1994), resting standing may contribute to heat production through postural muscle activity.

However, as for most other welfare issues, there is a lack of known visible indicators of thermal comfort per se, i.e., possible expression of positive emotions when environmental conditions are in an optimal range. Identifying such indicators would be particularly valuable for improving management practices, for instance by helping to determine optimal weather conditions for different categories of horses or informing decisions regarding the use of protective measures such as blankets. This is especially important as the repeated occurrence of such positive states may contribute to the development of chronic positive welfare (Boissy et al. 2007, Hausberger et al. 2019).

In this respect, acoustic signals constitute a promising, non-invasive tool as they reflect the internal state of the animal (e.g. Seyfarth and Cheney 2003; Briefer 2012). Some are consistently produced in positive situations (e.g., “laughing” sounds in rats, Panksepp and Burgdorf 2003, purring in Felids Peters 2002). Unvoiced acoustic signals (not produced by vocal folds) are of special interest as they are directly related to the breathing system, itself affected by emotions (Boyden 1994). Snorts in particular, a short sound produced during exhalation and through a vibration of nostrils, have received a growing interest as there is increasing evidence that they may reflect emotional states, beyond their presumed physiological origin of cleaning the nostrils (Jenikejew et al. 2025). Sneezes in wild dogs (*Lycaon pictus*) may be a pre-departure signal, reflecting arousal and/or preparing for next steps by clearing the nostrils (Walker et al. 2017). Snorts have been recorded in a wide range of taxa and been associated with a variety of arousal and valence states depending on species but also on the terminology used. In white rhinoceros (*Ceratotherium simum*) for example, snorts are produced in a relaxed state while feeding or resting (Policht et al. 2008; Linn et al. 2018; Jenikejew et al. 2020, 2025). Horses have an acoustic repertoire based on both voiced and unvoiced acoustic signals, some of which are associated with situations of negative valence (blows or snores produced in excitation/alarm contexts, e.g. Scopa et al. 2018; Contreras-Aguilar et al. 2019; groans in case of physical discomfort, Waring 2003), whereas others have a more ambiguous emotional « meaning » (nickers as close « contact calls », squeals during social encounters and whinnies as both social stress and reuniting signals, e.g. Briefer et al. 2015; Waring 2003; Yeon 2012). Horses also produce snorts which, as for white rhinoceros, constitute the most frequently emitted acoustic signal although snort production is highly dependent upon context. A series of recent studies on domestic horses have shown that snorts are associated with positive situations and constitute a likely candidate as an indicator of calm positive emotions (Stomp et al. 2018a). Thus, snort production is associated with more positive contexts (e.g. in pasture rather than in box, while feeding) and states (ears forward and never when ears backwards) (Stomp et al. 2018a). Moreover, within a same facility, individual variations have been observed: snort rate recorded during turn out in pasture was negatively correlated with indicators of compromised welfare such as a composite score of chronic stress, stereotypic behaviours or aggressiveness towards humans (Stomp et al. 2018a). Other evidences come from observations in the riding context, showing that snort production correlates negatively with rein tension (source of discomfort) in dressage horses (König von Borstel & Glißman 2014), increases in lame horses after treatment (Dyson et al. 2018) and is associated with relaxed times (i.e., when riders have less tight rein actions and horses are in a comfortable posture) during riding lessons (Stomp et al. 2020).

Therefore, we hypothesized that acoustic production, especially snort production, could constitute a window on how horses perceive the conditions of their outdoor housing and could help recognizing thermal comfort. In order to test this hypothesis, we recorded the sound production of non-pregnant broodmares over three in early Spring and examined possible correlates with meteorological factors (temperature, humidity, wind). Since Stomp et al. (2018a) reported anecdotal evidence of a strong increase of snort production in a group when changing pasture, we also paid attention to these events and the associated variations in food resources.

## Materials and methods

### Ethical note

The study was carried out in 2021 in accordance with the European Parliament and the European Union Council directive 2010/63/UE relative to the protection of animals used for scientific purposes and complied with the current French laws related to animal experimentation (Decree no. 2013 − 118 of 1 February 2013 and its five implementation orders (JO of 7th February 2013), integrated in the Code rural and the Code of the maritime fishing under no. R. 214 − 87 à R. 214 − 137). Since the study was only observational, it did not require any further ethical authorization. The owner of the horses (Institut du Cheval et de l’Equitation) and the facility manager allowed our research to be conducted on this site. Animal husbandry and care were under the management of the facility staff.

## Study site and population

The study was performed at the Experimental Station of Chamberet (Institut Français du Cheval et de l’Equitation) in France, between the 9th and 28th April 2021. Twenty adult breeding but not pregnant nor with foals, mares, aged 7 to 18 years, were observed. All mares were healthy with no history of respiratory problems and had been in the facility since birth. Over winter time, mares had been kept in three groups of 16, 2 and 2 respectively in large collective straw-bedded stalls with outdoor free access (bare ground) where they had hay and water *ad libitum*. From 7 April onwards, mares were turned out as a single group in a 2-ha pasture, with grass and water *ad libitum* where they remained 24 h/7 until next winter. Hedges and large trees constituted natural shelter but no artificial shelter was available. As part of routine management in the facility, each mare was marked with an individual number on the flank using a livestock pen allowing individual identification at distance. During the observation period, mares were moved to a new pasture three times as part of a pasture-rotation routine in this facility. (12th April= S2, 19th April= S7, 28th April= S13, Table 1)

**Table 1:** Observation sessions in which acoustic production was recorded using all-occurrence sampling

Session	Date
S1	09 April 2021
S2	12 April 2021
S3	13 April 2021
S4	14 April 2021
S5	15 April 2021
S6	16 April 2021
S7	19 April 2021
S8	20 April 2021
S9	22 April 2021
S10	23 April 2021
S11	26 April 2021
S12	27 April 2021
S13	28 April 2021

## Observation procedure

A single observer (LM) performed all the observations.

- Acoustic production: one two-hour session was performed daily, at different times of day (randomly distributed) between 8am and 5pm, up to five times a week. In total 13 observation sessions were performed (Table 1). Mares’ acoustic production was assessed using an *all occurrences* sampling (Altmann 1974). The observer was standing outside the pasture at the fence as close as possible to the horses. Thus, distances ranged between 5 and 20 m. This method allowed continuous monitoring of all 20 individuals while distance from the animal and background noise (wind, rain, tractors) would not have allowed an exhaustive recording of all sounds produced using standard acoustic equipment. The observer had been trained by an experienced senior scientist (MH) to recognize the different signals by ear using a large sample of sounds recorded previously by the research group until agreement reached 100%. These sounds are easily distinguished by ear (e.g., Stomp et al. 2018b). 

We used the terminology proposed by Waring (2003); Stomp et al. (2018a). Voiced (produced by vocal folds) signals included: whinnies – long-distance loud vocalizations; nickers – short-distance contact calls; squeals – high-pitched vocalizations involved in social interactions; groans – produced during physical discomfort. Unvoiced (i.e., produced by the passage of air through the nostrils) signals included: snores – very short raspy inhalation sounds produced in a low alert context (e.g., investigating a novel object or prior to emitting a blow); blows – short, very intense non-pulsed exhalations generally associated with vigilance or alarm postures; snorts – more or less pulsed sounds produced by nostril vibrations while expelling air, with a slightly longer duration compared to blows.

- Time-budget: mares’ time budget was assessed using a scan sampling method (Altmann 1974). A total of six sessions were conducted. Each session lasted 30 min, during which mares’ behaviours were recorded every 3 min. Two sessions (S1 and S5) took place immediately before the all-occurrences sampling sessions, while four sessions (S6, S10, S11 and S13) were conducted immediately after. This design aimed to ensure that behavioural and acoustic data were collected under similar meteorological conditions. In total, 1320 scans were recorded. For the time-budget sessions, the observer positioned himself on a higher point that allowed an unobstructed view of the numbers on the mares’ flanks. He used a voice recorder in order to record instantaneously the activity of each mare while he was scanning the group. Activities recorded were feeding, resting lying, resting standing (immobile with eyes partially closed, on 3 or 4 feet), exploration of the environment (e.g. sniffing), locomotion (only walk as no trot or canter was observed during the scans), maintenance (e.g. self -scratching, rolling), social interaction (e.g. allogrooming, sniffing, threat) observation (scanning of the environment with the neck horizontal or slightly above horizontal, with body immobile but mobile head, ears, neck, e.g. Rochais et al. 2016), gazing (immobile with fixed head, ears and neck at a horizontal to medium height) (Waring 2003). No vigilance (alarm) posture (high neck posture with fixed head, ears and neck, Kiley-Worthington 1976) was observed during these sessions.

## Measures of weather conditions and food resources

Data on weather conditions were obtained from the facility’s meteorological station (VantagePro, Davis Instruments) which provided the information on an hourly basis: temperature (in °Celsius), humidity (in %) and wind (in km/sec.). Since meteorological values recorded at the time of observation sessions were highly correlated with day values, mean day values were used for statistical analyses.

Moreover, routine data collected (on a five-point basis) by the facility staff on pasture characteristics were used, including grass height and botanical composition (categorized in grasses, legumes, and a “diverse” category including dandelion, dock, shepherd’s purse) to compare resource diversity just after horses entered a pasture and just before they left it, as an indirect estimate of horses’ feeding preferences. The measurements were taken on parallel transects covering the entire pasture (approximately 200 sample points). The botanical diversity was measured on three 25-m-long fixed transects with one sample location every meter. At each sample location,

five points (from zero, absence; to five, exclusive presence) were distributed among the different plant species. The specific contribution of each plant species at the transect scale was given in%): Plant identification followed (Tutin et al. 1964) and, for Festuca species, (Kerguélen and Plonka 1989). The plant species were then split into grasses, legumes and « diverse ».

### Statistical analysis

Horses produced very few voice signals over the observation period and most sounds produced were snorts (see results).

To evaluate the influence of environmental and contextual factors on snorts’ frequency, we fitted generalized linear mixed models (GLMMs) with a negative binomial error distribution using the package lme4 in R (v.4.3.2). The response variable was number of snorts. Residuals were inspected for patterns and overdispersion, confirming that the negative binomial distribution adequately. The full model included five fixed effects: day temperature (Temperature), day humidity (Humidity), wind speed (Wind), time of day (coded as “Morning” or “Afternoon”), and whether the recording occurred just after entry or just before exit from the current pasture (Entry_Exit, Entrance vs. Exit). Two random intercepts were included to account for repeated measures: individual identity (Individual) and observation session (Session). To check for collinearity among the fixed effects, variance inflation factors (VIFs) were calculated using the performance package. All VIF values were below 2.5, indicating no problematic multicollinearity among predictors. To assess the contribution of the fixed effects, we compared this full model to a null model that included only the random intercepts (individual and session) and no fixed predictors. A likelihood ratio test was conducted to compare the two models.

To assess whether temperature and humidity differed between morning and afternoon sessions, we performed a Welch two-sample t-test. Moreover, we performed a Principal Component Analysis (PCA) to explore the multivariate relationships between behavioral variables, acoustic indicators (snorts), and environmental parameters recorded across eight observation sessions. The PCA included 15 quantitative variables describing behavior (feeding, resting lying, exploration of the environment, observation, gazing, locomotion, maintenance, resting standing, and social interaction), acoustic features (mean, median, and standard deviation of snorts), and environmental conditions (temperature, humidity, and wind). Session date and number of sessions were retained as metadata and were not included in the analysis. Prior to the PCA, all variables were centered and scaled to unit variance. The analysis was performed in R version 4.2.1 using the packages tidyverse and factoextra. Eigenvalues and the proportion of variance explained were used to interpret the principal components.

## Results

Over the whole observation period, 2 887 sounds were recorded : 2 695 snorts (produced by 20/20 horses, $\:\stackrel{-}{\boldsymbol{X}}$ ± se=134,7 ± 29,3 |0–205]), 58 squeals (16/20 horses, $\:\stackrel{-}{\boldsymbol{X}}$ ± se = 2,9 ± 0,72, [0–4]), 56 nickers (16/20 horses, $\:\stackrel{-}{\boldsymbol{X}}$ ± se = 2,8 ± 0,63, [0–3]), 41 snores (12/20 horses, $\:\stackrel{-}{\boldsymbol{X}}$ ± se = 2,05 ± 0,58, [0–5]), 9 sighs (8/20 horses, $\:\stackrel{-}{\boldsymbol{X}}$ ± se = 0,45 ± 0,13, range: 0–2), 5 blows (1/20 horse), 3 whinnies (1/20 horse) and 1 groan (1/20 horse).

Almost all squeals (45/58) were recorded on the first observation day, i.e. just after the 3 winter groups of mares had been released together. All nickers were recorded during 2 sessions, one when a new group of mares had been released on a nearby pasture, and the other when a tractor (used for carrying hay) passed close-by. The other sound types were produced very occasionally over the whole observation period.

Thus, the snorts were the only signal that was produced by all mares and at a significantly high level. Therefore, the subsequent analyses have focused on snort production.

## Snort production and meteorological data

Snort production showed marked variations across sessions, ranging from a minimum of 5 snorts per individual (S2 on 12th April, i.e. less than 200 snorts at the group level) and a maximum of 30 snorts/individual (S11 on 27th April, i.e. around 1 500 at the group level).

**Fig. 1:**
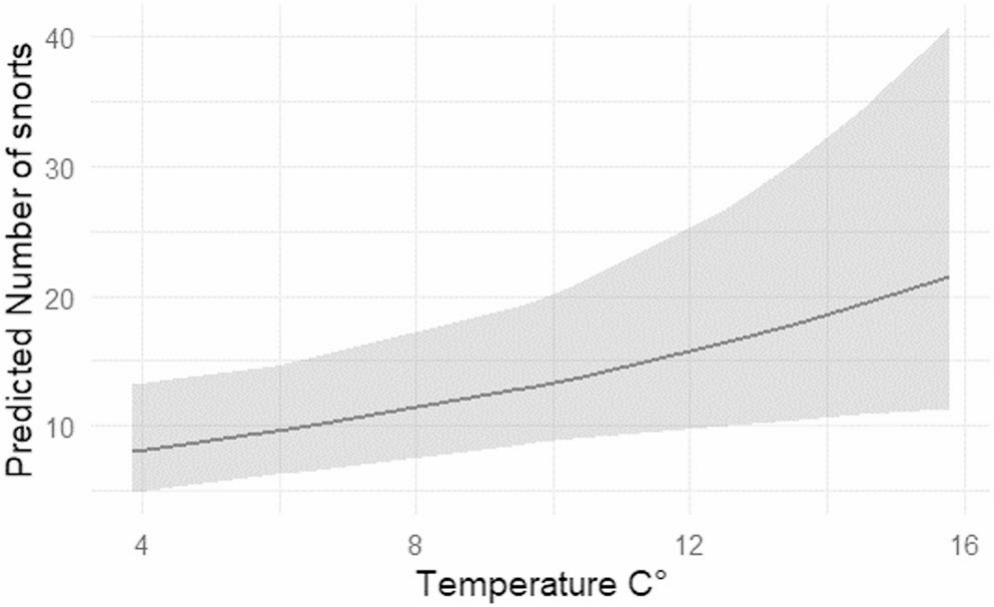
Predicted number of snorts as a function of daily temperature. The solid line represents the predicted mean number of snorts from the GLMM, and the shaded area indicates the 95% confidence interval

**Fig. 2:**
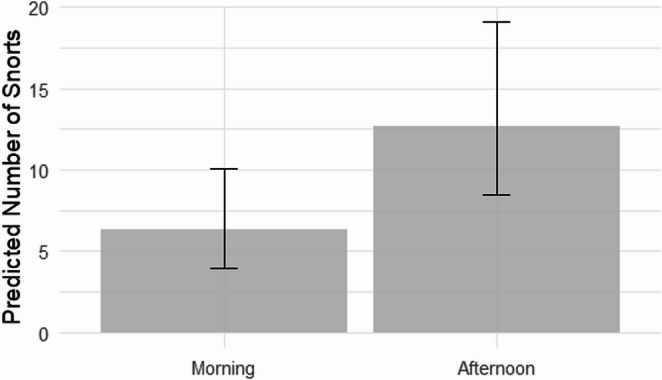
Predicted number of snorts depending on time of day. Bars represent the predicted mean number of snorts for morning and afternoon sessions from the GLMM, and error bars indicate the 95% confidence interval

**Fig. 3:**
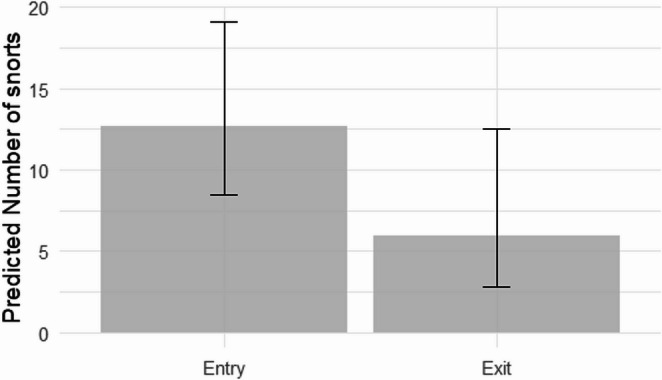
Predicted number of snorts depending on the recording context (Entry vs. Exit). Bars show the predicted mean number of snorts from the GLMM, and error bars represent the 95% confidence interval

Snort frequency was significantly influenced by environmental and contextual factors (Table 2; Suppl. Table 1). Daily temperature showed a positive effect on the number of snorts, with an estimated 8.5% increase in snort frequency for each 1 °C increase in temperature (IRR = 1.085, *p* = 0.016; Table 2; Fig. 1). Moreover, snort number was significantly lower during morning sessions compared to afternoon sessions, corresponding to an approximately 50% reduction (IRR = 0.499, *p* = 0.020; Table 2; Fig. 2). Similarly, recordings made just before exiting the pasture were associated with significantly fewer snorts than those made immediately after entry, with an estimated reduction of 53.5% (IRR = 0.465, *p* = 0.012; Table 1; Fig. 3). Wind speed and humidity did not significantly affect snort number (wind: IRR = 1.113, *p* = 0.113; humidity: IRR = 1.000, *p* = 0.986; Table 2). A likelihood-ratio test comparing the full model with a null model including only random effects indicated that the inclusion of environmental and contextual predictors significantly improved model fit (χ² = 18.19, df = 5, *p* = 0.0027). Random effects revealed moderate variability in snort frequency among individuals and observation sessions. Concerning whether environmental and behavioural factors could explain the difference in snort production between morning and afternoon sessions we found that while temperature did not differ (*p* = 0,80), humidity was significantly higher in the morning (*p* < 0.001), suggesting a potential climatic contribution to this effect.

**Table 2:** Effects of environmental and contextual predictors on horse snort frequency. Estimated coefficients were exponentiated to obtain incidence rate ratios (IRR), representing the multiplicative change in snort number for a one-unit increase in continuous predictors or relative to the reference level for categorical predictors. Percent change (% change) corresponds to (IRR − 1) × 100. Reference levels: Afternoon for Time_of_day and Enter for Entry_Exit

Predictor	β	SE	IRR	% change	*p*
Temperature	0.082	0.034	1.085	8.5	0.016
Humidity	0	0.01	1	0	0.986
Wind	0.107	0.067	1.113	11.3	0.113
Time_of_day (Morning)	−0.696	0.299	0.499	−50.1	0.02
Entry_Exit (Exit)	−0.766	0.3	0.465	−53.5	0.011

**Fig. 4:**
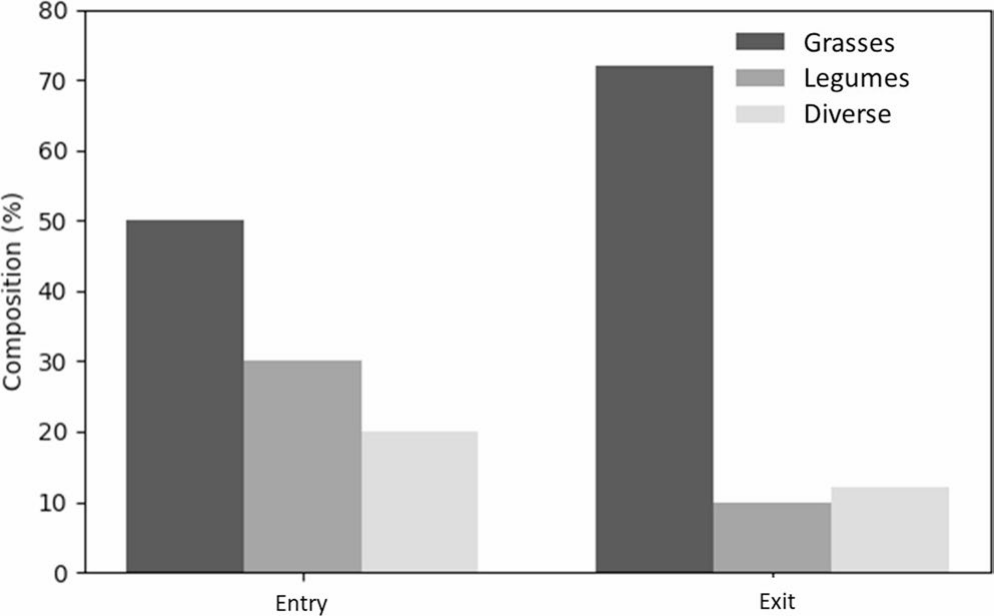
Pasture composition at two management stages: immediately after pasture entry and prior to pasture transfer. Pasture composition differed significantly between pasture states (χ² test, *p* < 0.0001)

Grass height did not correlate with snort production (Table 3), but pasture composition differed between horses’ entry and exit from the pastures: resource diversity was higher just after entry in a new pasture (50% grass, 30% legumes, 20% diverse) than before exit (72% grass, 10% legumes, 12% diverse) (ꭓ² test: *p* < 0,0001) Fig. 4.

**Table 3:** Mean grass height just after entrance of the horses in a pasture and just before they were transferred to a new one and median and mean production of snorts

Pasture	Entry-Exit	cycle	Snorts Mean	Snorts Median	Grass height mean (cm)	Grass height SD (cm)
P0	Entry	1	15.35	10	12.60	NA
P0	Exit	1	2.55	2	5.50	NA
P1	Entry	1	9.3	6	14.01	NA
P1	Exit	1	4.75	3	15.16	1.20
P2	Entry	1	8.8	5	27.74	2.43
P2	Exit	1	4.4	4	19.22	4.57
P3	Entry	1	26.15	18	14.22	0.94
P3	Exit	1	1.35	0.5	7.22	1.01

## Weather conditions, snort production and behavioural activities

**Fig. 5:**
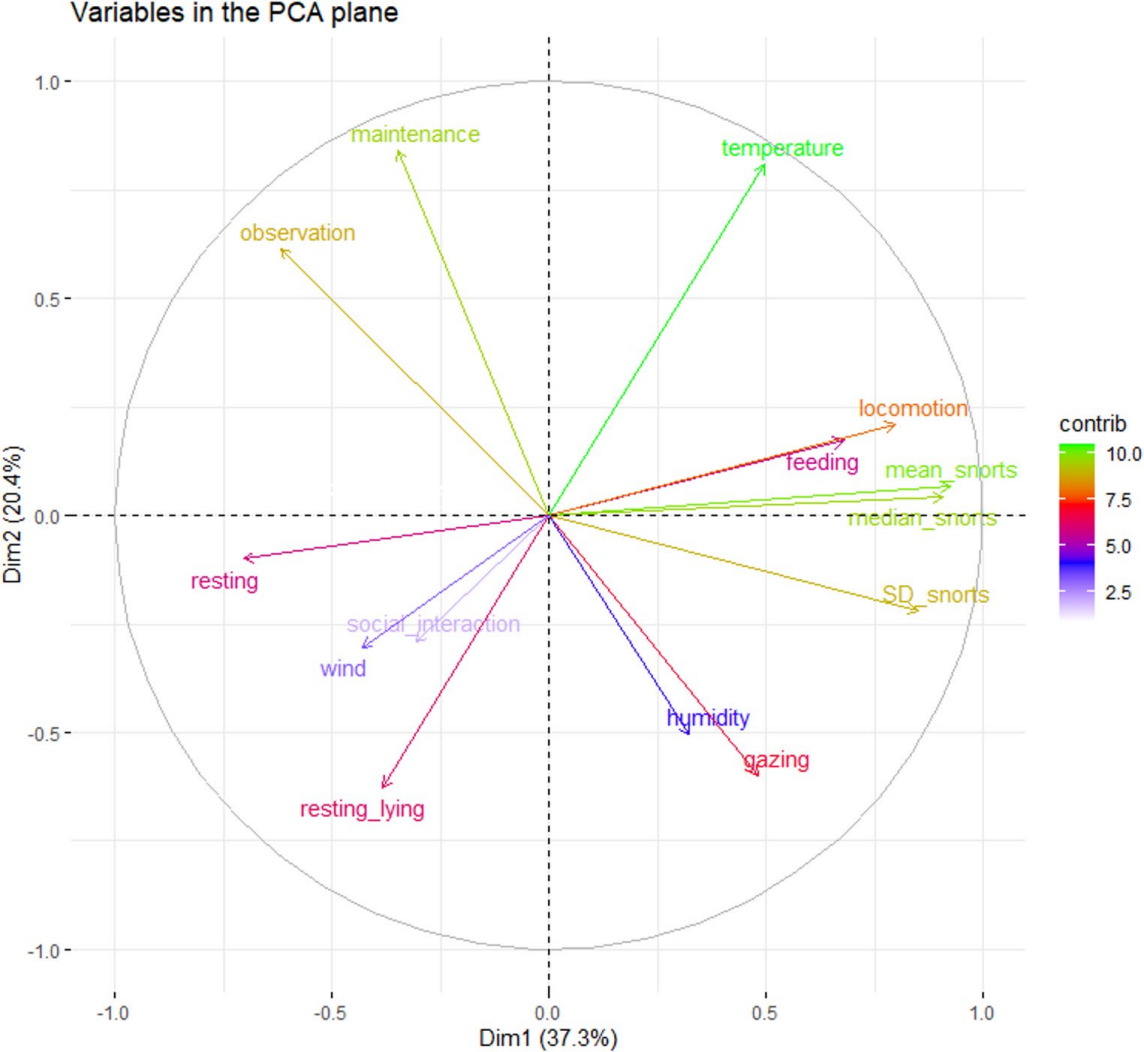
Principal component analysis (PCA) biplot of behavioral and environmental variables. Arrows represent variable loadings on the first two principal components, which together explain 57.7% of the total variance

The relationships between behavioural variables, acoustic indicators and environmental parameters were further explored using a principal component analysis (Fig. 5; Table 4). The relative contributions of variables to the first three principal components are reported in Table 4 while full eigenvalues and loadings are provided as supplementary material (Tables S2 and S3). The PCA revealed that the first two principal components accounted for 57.7% of the total variance in the dataset. PC1 explained 37.3% and PC2 explained 20.4% of the variance. Subsequent components contributed progressively smaller proportions (PC3 = 14.7%, PC4 = 12.2%, PC5 = 8.8%), indicating that substantial proportion of the structure of the data was captured by the first dimensions. PC1 primarily represented a gradient from higher to lower behavioural activity. Sessions with higher scores on this axis were characterized by increased locomotion, feeding, and higher snort production (mean, standard deviation SD, and median), together with higher temperatures. In contrast, the opposite end of PC1 was associated with more static behaviours such as resting standing and gazing. PC2 captured a second behavioural–environmental axis opposing maintenance and observation behaviours, together with temperature, to more passive behaviours such as resting lying and gazing, and higher humidity. This suggests that sessions characterized by increased maintenance or observation behaviour differed from those dominated by static behaviours occurring under more humid conditions. Although snort production was not associated with PC3, this component, which explained a smaller proportion of the variance, suggested an association between social interactions, higher wind, and resting behaviours. However, this axis should be interpreted cautiously given its lower explanatory power.

**Table 4:** Only variables contributing ≥ 5% to at least one of the two components are shown

Feeding	PC1 (%)	PC2 (%)	PC3 (%)
	8.25	0.98	17.3
Resting lying	2.61	12.88	8.11
Exploration	1.41	0.02	0.23
Observation	6.83	12.34	0
Gazing	4.18	11.74	2.02
Locomotion	11.42	1.43	3.9
Maintenance	2.16	23.06	1.72
Resting standing	8.84	0.33	14.79
Social interaction	1.68	2.74	28.13
Mean snorts	15.31	0.15	0
SD snorts	12.93	1.57	0.01
Median snorts	14.77	0.06	0.51
Temperature	4.43	21.36	0.02
Humidity	1.87	8.31	1.54
Wind	3.31	3.03	21.71

## Discussion

Observations of acoustic production of adult horses living in a stable group on pasture confirmed earlier studies showing that (e.g., Waring 2003), overall, acoustic production is low in this context, except for snorts. Snort production varied markedly across sessions and was highly positively correlated with daily ambient temperature in this temperate early Spring situation. There were no correlates between snort production and humidity although both variables had opposite loadings on one PCA axis. Overall, the PCA combining time-budget, snort production, and meteorological variables indicated that, in these early spring conditions, warmer days (around 20 °C) were associated with more active behaviours (feeding, locomotion, observation), whereas cooler (< 10 °C) and more humid conditions were associated with more static behaviours (resting standing, gazing) and lower snort production. Finally, snort production was higher after entry into a new pasture with more diversified resources.

These findings are consistent with earlier observations showing that snort production is associated with relaxed positive situations. Snort production co-occurred with other behavioural activities in environmental conditions characterized by moderately high temperature and limited adverse factors such as wind or humidity. Indeed, in these conditions, together with higher snort production, the predominant activities observed were feeding, calm locomotion and observation behaviours (scanning of the environment). Interestingly, a recent study has shown that these calm “mobile” activities occupy a large part of the time budget in horses that are in a good welfare state and are experiencing more appropriate housing and working conditions (Hausberger et al. in rev.). In this same study, snort production was positively correlated with the time spent in feeding, observation and positive social interactions, reflecting how snorts were closely related to positively valanced activities. Observation behaviours, which are also more present in horses in better dorsal health (Rochais et al. 2016), appear, in this Hausberger et al. (in rev.) study as in the present study, to occur in contexts that are opposite to those where fixed attention (gazes) occur. Here gazing was associated with higher humidity. Overall, static behaviours, such as gazing and resting standing, occurred more when weather conditions were probably perceived as less favorable by horses (low temperatures, wind, humidity) while in Hausberger et al. (in rev.) study, they appeared more when feeding or riding conditions were less appropriate (see also Raspa et al. 2020). Here, the mares had all their basic needs fulfilled, as they had permanent access to free movement, food and water ad libitum, were kept in a stable social group (Krueger et al. 2021) and were not subjected to work-related constraints, which prevented them from experiencing any possible related stress. This increased further the chances that horses produce snorts (Stomp et al. 2018a). White rhinoceros also produce mostly snorts during feeding and slow locomotion (Policht et al. 2008; Linn et al. 2018). Moreover, transfers of mares towards new pastures with more diversified resources were associated, like in Stomp et al. (2018a)’s report, with an increase of snort production. Comparisons of pasture composition at entry and exit confirmed that resource diversity decreased over the period of use. In natural conditions and when given the opportunity, horses consume up to 50 different types of plants that they actively select  (van den Berg et al. 2015). In the same line, experimental studies have shown that, if given the choice, horses prefer a more diversified roughage to a single type of roughage (e.g., Goodwin et al. 2002).

The low snort production and higher time spent in static behaviours such as resting (mainly standing) or gazing (fixed attention to the environment) observed when temperatures were lower, and especially if there was high humidity may indicate that horses were experiencing thermal discomfort (as a strategy to save energy (Brinkmann et al. 2014). Brinkmann et al. (2014) observed that the metabolism rate of Shetland ponies was reduced during resting which is in accordance with observations on red deers and Przewalski horses that reduce energy expenditure in winter (Arnold et al. 2004, 2006). Outdoor housed ponies show a larger locomotion time span in summer than in winter (Brinkmann et al. 2014). Although temperatures did not go below 5 °C during our study period, it is thus quite possible that the mares experienced some thermal discomfort. Thermal perception is highly subjective and related to horse’s characteristics and experience. Here, the animals were all from the same sex and breed, which may explain the major impact of environmental factors at the population level. Most studies questioning thermal comfort in horses have been made in extreme climatic conditions, which makes comparisons with our study difficult. Nevertheless, when Mejdell et al. (2016, 2019) « questioned » horses through conditioning about whether they wanted to have a rug or not, horses, despite their experience of very low temperatures, requested a rug at 6 °C when it was rainy. Snoeks et al. (2015) found an increase in artificial shelter used when temperatures decreased to 7 °C and under in horses living outdoors in Belgium. In experimental studies, lowest comfort temperature varies from − 15 °C in quarter horses winter acclimatized (McBride et al. 1985, Cymbaluk and Christison 1989) to + 5 °C in standardbred trotters acclimatized to 15–20 °C (Morgan 1998). The study mares had spent winter in collective stalls with an outdoor access, which means they were within three full walls, with straw bedding and hay. A mere unheated shelter reduces the climatic energy demand (MacCormack & Bruce 1991) while roughage provides energy, reducing the effects of the cold temperature on horses’ metabolism (Brinkmann et al. 2014). Therefore, mares may have subjectively perceived the moderately low temperatures when housed outdoors as a source of discomfort. We found no interaction between temperatures and humidity in the factors influencing snort production. Neither humidity nor wind seemed to influence snort production. However, there were overall less snorts when there was more humidity. This suggests horses may be primarily sensitive to temperature, with secondary factors such as humidity modulating their perception, which would fit with the observations performed in extreme cold climates (Mejdell et al. 2020). Although wind was not associated with snorts, it loaded on the PCA 3 axis with social interactions. Wind may trigger excitation and as a consequence increase social interactions and even play in adult horses (Blois-Heulin et al. 2015; McDonnell & Poulin 2002).

The strong correlation between snort production and environmental temperature is intriguing, but there were no temperatures above 20 °C. Horses may appreciate such moderately high temperatures. Bahaloudis et al. (2024) found that feral horses living in northern Greece selected areas with high solar radiation (rather than forested areas for shelter). Future studies should aim at investigating behaviour and snort production at higher temperatures, particularly approaching heat-induced thermal discomfort, where a decrease in snort production would be expected.

The rather short time span is one limitation of this study, as temperatures thus remained in a moderate range. Also, this study would need to be repeated in different facilities and climates, including in feral populations. Finally, we were not able to measure pollen dissemination and possible insects. However, in this region, grass pollen occurs mainly from May on, i.e., after the study period. Moreover, earlier studies clearly show that snort production cannot be fully explained by its mere hygienic function, whether in horses on in other species (Stomp et al. 2018a; Hausberger 2019).

In conclusion, among the different acoustic signals produced by horses, snorts appeared as a particularly promising indicator of comfort in outdoor conditions. Snort production increased with higher ambient temperatures, was higher in more favorable pasture conditions (i.e., after entry into a new pasture with more diverse resources), and was associated with more active behavioural states. Conversely, lower snort production was observed under cooler conditions and in association with more static behaviours. Taken together, our results suggest that horses may have experienced thermal comfort at temperatures between 15 and 20 °C and some discomfort at temperatures under 10 °C. They suggest that snort production may be a simple, easy and fruitful way of identifying whether the outdoor conditions offered are perceived positively by the horses, and thus could be a useful tool for assessing and improving welfare conditions. By linking acoustic communication to climatic conditions and resource availability, our study provides insights into the mechanisms through which environmental variation shapes animal behaviour and emotional states. More broadly, it contributes to the understanding of animal–environment interactions and highlights acoustic signals as functional indicators of internal states, a topic of broad relevance across taxa and disciplines.

## Supplementary Information

Below is the link to the electronic supplementary material.

Supplementary Material 1 (DOCX 20.4 KB)

## Data Availability

The entire dataset and comprehensive statistical code have been deposited in the Figshare data repository at the following address: https://figshare.com/s/0c4537107c90093bc1cc.
