# Supplementary material for: Could snort production reflect comfort in horses kept outdoors? A first study
Source: Naturwissenschaften. 2026 Jun 30;113(4):79. doi: 10.1007/s00114-026-02113-3 (PMC13320096; doi:10.1007/s00114-026-02113-3)
Supplement: Supplementary file 1 — Supplementary Material 1 (DOCX 20.4 KB) [file 114_2026_2113_MOESM1_ESM.docx]

**Table S1** Fixed‐effect estimates from the negative binomial GLMM assessing the influence of environmental and contextual variables on the number of snorts in horses. Number of observations = 260; individuals = 20; sessions = 13. Estimates (β), standard errors (SE), z-values, and associated p-values are reported

| **Predictors** | **ß** | **SE** | **t value** | **p** |
| --- | --- | --- | --- | --- |
| (Intercept) | 1.445 | 0.870 | 1.660 | 0.097 |
| **Temperature** | 0.082 | 0.034 | 2.412 | **0.016** |
| Humidity | 0.001 | 0.009 | 0.017 | 0.986 |
| Wind | 0.107 | 0.067 | 1.585 | 0.113 |
| **Time_of_day_Morning** | -0.696 | 0.299 | -2.325 | **0.020** |
| **Entry_Exit_Exit** | -0.765 | 0.299 | -2.553 | **0.012** |

The bold values indicate statistically significant results.

**Table S2** Eigenvalues and percentage of variance explained by each principal component, and cumulative variance for each principal component obtained from the PCA. The first two components account for 57.7% of the total variance

|  | **Eigenvalue** | **Variance percent** | **Cumulative Variance percent** |
| --- | --- | --- | --- |
| **Dim.1** | 5.59E+00 | 3.73E+01 | 37.29963 |
| **Dim.2** | 3.06E+00 | 2.04E+01 | 57.68774 |
| **Dim.3** | 2.20E+00 | 1.47E+01 | 72.33979 |
| **Dim.4** | 1.84E+00 | 1.22E+01 | 84.58166 |
| **Dim.5** | 1.32E+00 | 8.77E+00 | 93.34849 |
| **Dim.6** | 6.58E-01 | 4.39E+00 | 97.73787 |
| **Dim.7** | 3.39E-01 | 2.26E+00 | 100 |
| **Dim.8** | 6.27E-32 | 4.18E-31 | 100 |

**Table S3** Behavioral and Environmental Variable Contributions Across Principal Components (PC1–PC8)

|  | **PC1** | **PC2** | **PC3** | **PC4** | **PC5** | **PC6** | **PC7** | **PC8** |
| --- | --- | --- | --- | --- | --- | --- | --- | --- |
| Feeding | 0.083 | 0.010 | 0.173 | 0.004 | 0.050 | 0.078 | 0.006 | 0.381 |
| Resting lying | 0.026 | 0.129 | 0.081 | 0.081 | 0.000 | 0.016 | 0.359 | 0.108 |
| Exploration environnement | 0.014 | 0.000 | 0.002 | 0.363 | 0.141 | 0.096 | 0.002 | 0.007 |
| Observation | 0.068 | 0.123 | 0.000 | 0.077 | 0.054 | 0.036 | 0.011 | 0.069 |
| Gazing | 0.042 | 0.117 | 0.020 | 0.045 | 0.195 | 0.035 | 0.002 | 0.002 |
| Locomotion | 0.114 | 0.014 | 0.039 | 0.084 | 0.000 | 0.113 | 0.006 | 0.231 |
| Maintenance | 0.022 | 0.231 | 0.017 | 0.001 | 0.001 | 0.190 | 0.026 | 0.001 |
| Resting | 0.088 | 0.003 | 0.148 | 0.040 | 0.061 | 0.007 | 0.036 | 0.109 |
| Social interaction | 0.017 | 0.027 | 0.281 | 0.018 | 0.037 | 0.151 | 0.066 | 0.002 |
| Mean snorts | 0.153 | 0.001 | 0.000 | 0.004 | 0.090 | 0.019 | 0.002 | 0.001 |
| SD snorts | 0.129 | 0.016 | 0.000 | 0.002 | 0.124 | 0.080 | 0.023 | 0.014 |
| Median snorts | 0.148 | 0.001 | 0.005 | 0.006 | 0.077 | 0.063 | 0.020 | 0.041 |
| Temperature | 0.044 | 0.214 | 0.000 | 0.029 | 0.001 | 0.005 | 0.119 | 0.001 |
| Humidity | 0.019 | 0.083 | 0.015 | 0.202 | 0.133 | 0.000 | 0.179 | 0.025 |
| Wind | 0.033 | 0.030 | 0.217 | 0.043 | 0.035 | 0.110 | 0.143 | 0.007 |
